# Reinterventions following laparoscopic cholecystectomy and bile duct exploration. A review of prospective data from 5740 patients

**DOI:** 10.1007/s00464-021-08568-x

**Published:** 2021-06-02

**Authors:** Hwei Jene Ng, Ahmad H. M. Nassar

**Affiliations:** 1 Laparoscopic Biliary Surgery Service, University Hospital Monklands, Airdrie, Scotland, UK; 2 University Hospital Monklands, Airdrie, Scotland, ML6 0JS UK

**Keywords:** Laparoscopic cholecystectomy, Reintervention, Complications, Bile duct exploration

## Abstract

**Background:**

Complications following laparoscopic cholecystectomy (LC) and common bile duct exploration (CBDE) for the management of gallstones or choledocholithiasis impact negatively on patients’ quality of life and may lead to reinterventions. This study aims to evaluate the causes and types of reintervention following index admission LC with or without CBDE.

**Methods:**

A prospectively maintained database of LC and CBDE performed by a single surgeon was analysed. Preoperative factors, difficulty grading and perioperative complications requiring reintervention and readmissions were examined.

**Results:**

Reinterventions were required in 112 of 5740 patients (2.0%), 89 (1.6%) being subsequent to complications. The reintervention cohort had a median age of 64 years, were more likely to be females (*p* < 0.0023) and to be emergency admissions (67.9%, *p* < 0.00001) with obstructive jaundice (35.7%, *p* < 0.00001). 46.4% of the reintervention cohort had a LC operative difficulty grade IV or V and 65.2% underwent a CBDE. Open conversion was predictive of the potential for reintervention (*p* < 0.00001). The most common single cause of reintervention was retained stones (0.5%) requiring ERCP followed by bile leakage (0.3%) requiring percutaneous drainage, ERCP and relaparoscopy. Relaparoscopy was necessary in 17 patients and open surgery in 13, 6 of whom not resulting from complications. There were 5 deaths.

**Conclusion:**

This large series had a low incidence of reinterventions resulting from complications in spite of a high workload of index admission surgery for biliary emergencies and bile duct stones. Surgical or endoscopic reinterventions following LC alone occurred in only 0.8%. The most common form of reintervention was ERCP for retained CBD stones. This important outcome parameter of laparoscopic biliary surgery can be optimised through early diagnosis and timely reintervention for complications.

## Background

Complications following laparoscopic cholecystectomy and intraoperative cholangiography (IOC) with or without common bile duct exploration (CBDE) pose various diagnostic and management challenges to the surgeon and impact negatively on the patient’s quality of life. The nature and severity of a complication will dictate the urgency with which it is to be investigated and determine whether conservative management will be sufficient to remedy that complication. Occasionally, reintervention is necessary to either improve the outcome or to avoid serious life threatening consequences.

Reintervention following LC and CBDE ranges from placing one suture to control port site bleeding to laparotomy and biliary reconstruction for a bile duct injury. Although most studies addressing biliary surgery report their complications and reinterventions, the last large study focusing on reinterventions as an outcome parameter following LC was published in 2002 [1].

We aim to review the causes of reinterventions following index admission LC, IOC and, when necessary, CBDE in a large series performed by a single surgeon providing a specialised acute biliary service with a high emergency workload. The secondary aim was to report the causes of reinterventions in patients who have not suffered any complications, a group that is usually overlooked in the literature.

## Methods

A prospectively maintained database of emergency and elective patients undergoing LC with or without CBDE performed by a single surgeon (AHMN) or by his trainees under direct on table supervision between 1992 and 2020 was reviewed. Data on patient demographics, type of admission, clinical presentation, admission to referral and referral to surgery intervals, operative difficulty grading, open conversion, perioperative complications, reinterventions, readmissions within one year and the 30-day mortality were analysed. The operative difficulty grade was based on the Nassar Scale [2–4]. This is a five grade system of describing the operative findings according to the adhesions around the gallbladder, the state of the cystic pedicle and the condition of the gallbladder. The objective assessment of operative complexity facilitates audit, research, assessment of training and comparison between studies. The referral pathways and operative techniques have been described in detail in previous studies [5–8].

Informed consent was obtained from all patients with emphasis on the specialisation of the unit with regard to the management of suspected bile duct stones. Ethical approval was not required as the management protocols were in line with the guidelines and recommendations of national and international societies.

Statistical analysis: Categorical variables are expressed as *n* and percentage (%) and continuous as mean ± standard deviation. Student’s t test was used for continuous variables and Chi-square or Fisher's Exact test for categorical variables. *P* value of < 0.05 was considered statistically significant. All analyses were performed using IBM SPSS 22.

## Results

Of the 5740 patients, 112 (2.0%) patients required 157 postoperative reinterventions using different modalities. The complication rate in the whole series was 4.9%, but only 89 patients (1.6%) had suffered operative or postoperative complications requiring 110 reinterventions. 23 patients (0.4%) needed 47 reinterventions indicated by reasons other than complications. 39 reinterventions occurred in 4422 patients who underwent LC without CBDE (0.8%). Of these surgical reintervention was only resorted to in 10 patients (0.2%).

The median age was 64 years (21–88 years) and the male to female ratio was 1:1.6. 67.9% of this patient cohort was emergency admissions. The primary admission diagnosis was jaundice in 35.7% and 32 (28.6%) had previous emergency biliary admissions, most before being referred to the biliary unit. The original operation was performed within five days of admission in 86.6% of the patients. Demographic characteristics and preoperative data of this cohort are shown in Table 1.

**Table 1:** Demographic and preoperative data of patients needing postoperative reintervention

Preoperative characteristics	Reinterventions (*n* = 112) (%)
Gender	
Male	43 (38.4%)
Female	69 (61.6%)
Type of admissions	
Emergency	76 (67.9%)
Electives	36 (32.1%)
Source of referral	
Other surgical teams	52 (46.4%)
Biliary team	37 (33.0%)
Other hospitals	17 (15.2%)
Physicians	6 (5.4%)
Primary admission diagnosis	
Jaundice	40 (35.7%)
Chronic biliary colic	33 (29.5%)
Cholangitis	12 (10.7%)
Acute biliary colic	13 (11.6%)
Acute pancreatitis	7 (6.3%)
Acute cholecystitis	7 (6.3%)
Diagnosis of previous biliary admissions	*N* = 32 (28.6%)
Jaundice	10 (31.3%)
Acute pain	10 (31.3%)
Acute cholecystitis	10 (31.3%)
Acute pancreatitis	2 (6.3%)
Interval between referral/ admission to surgery (day)	
0–1	68 (60.7%)
2–5	29 (25.9%)
6–10	10 (8.9%)
> 10	1 (0.9%)
No records	4 (3.6%)

46.4% of the patients requiring reintervention had operative difficulty grades IV or V (compared to only 15.7% in the rest of the series). Most LC/CBDEs were performed by the consultant. In this cohort, 65.2% had CBDE; 51 (69.9%) via choledochotomy and 22 (30.1%) by transcystic exploration. The perioperative findings are summarised in Table 2.

**Table 2:** Perioperative characteristics of patients needing postoperative reintervention

Operative Characteristics	Patients (*n* = 112) (%)
Operative difficulty grade	
Grade 1	13 (11.6%)
Grade 2	26 (23.2%)
Grade 3	21 (18.8%)
Grade 4	40 (35.7%)
Grade 5	12 (10.7%)
Operator	
Consultant	93 (83.0%)
Trainee	19 (17.0%)
CBD exploration	
Yes	73 (65.2%)
No	39 (34.8%)
Conversion to open	5 (4.5%)
Length of surgery, median (range)	120 (32–390) minutes
Mortality	5 (4.5%)
Length of hospital stay, median (range)	16 (1–160) days
Readmissions	27 (24.1%)
Number of episodes, median (range)	2 (1–8)
Presentation to resolution, median (range)	5 (1–56) weeks

It is interesting that acute cholecystitis and gallbladder empyema were not significant risk factors for reintervention, while the presence of Hartmann’s pouch stones or contracted gallbladder were significant.

Five patients (4.6%) in this reintervention cohort had been converted from laparoscopic to open surgery: 3 due to dense adhesion and 2 due to multiple large impacted CBD stones. Three subsequently underwent ERCP for retained CBD stones and two needed percutaneous drainage of subphrenic abscesses.

**ERCP** was the most common postoperative biliary reintervention. 64 (1.1%) patients required a total of 97 ERCPs post LC or CBDE. 15 of these patients did not have ERCP as a result of complications: eight were in the course of managing pancreatic cancers/ periampullary lesions discovered on IOC, five for the management of benign biliary strictures and two for the removal of stents. ERCP was required in 26 patients to extract retained CBD stones (21 after CBDE and 5 after LC) and in 2 for suspected CBD stones. 13 ERCPs were needed for the management of bile leakage and one to remove a biliary drain that had retracted into the abdomen. Other causes are summarised in Table 3.

**Table 3:** Causes for surgical reintervention

Reintervention	Causes	Clavien-Dindo Classification	Number (*N* = 157)
ERCP	Retained stone after CBDE	Grade 3a	21 + 12^**^
	Retained stone after LC	Grade 3a	5
	Pancreatic cancer^*^	Grade 3a	7
	Periampullary tumour^*^	Grade 3a	3
	Benign stricture^*^	Grade 3a	5 + 9^**^
	Bile leak	Grade 3a	13
	Suspected retained stone	Grade 3a	2
	Removal of stent^*^	Grade 3a	14
	Biliary drain related	Grade 3a	1
	Equivocal cholangiogram	Grade 3a	2
	Pancreatic necrosis	Grade 3a	1
	Post-op jaundice	Grade 3a	1
	Mirizzi Syndrome III	Grade 3a	1
Relaparoscopy	T-tube related	Grade 3b	5
	Bile leak	Grade 3b	5
	Bleeding from omentum	Grade 3b	2
	Removal of drain	Grade 3b	1
	Restenting CBD	Grade 3b	1
	Perforated duodenal ulcer	Grade 3b	1
	Abscess collection	Grade 3b	1
Relaparoscopy with ileostomy formation	Colonic leak from cholecystocolic fistula	Grade 3b	1
Biliary bypass	Mirizzi Syndrome IV^*^	Grade 3b	1
	Mirizzi Syndrome III	Grade 3b	1
Biliary reconstruction	CBD injury	Grade 3b	2
	Gallbladder cancer^*^	Grade 3b	1
Whipple’s procedure	Periampullary tumour^*^	Grade 3b	2
	Pancreatic cancer^*^	Grade 3b	2
Laparotomy	Mesenteric ischaemia	Grade 5	2
	Perforated large bowel	Grade 5	2
Percutaneous drainage	Abscess collection	Grade 3a	9
	Bile leak	Grade 3a	8
Gastroscopy	Removal of stent^*^	Grade 3a	2
	T-tube related	Grade 3a	1
Suture under local anaesthesia	Port site bleeding	Grade 3a	5
Reventilation	Chest infection	Grade 4a	2
	Hypoxia	Grade 4a	1
Chest drain	Pneumothorax	Grade 3a	1
Embolisation under interventional radiology	Hepatic artery aneurysm^*^	Grade 5	1

*Reinterventions not due to postoperative complications (47 reinterventions in 23 patients)

**Repeated procedure (12 to remove further retained CBD stone, 9 to remove/change stent for benign stricture)

**Further surgical intervention** was necessary in 30 (0.5%) patients: 17 relaparoscopies and 13 by open surgery. Reoperation by open surgery included 6 patients who had not had any complications: five with incidental pancreatic, periampullary and gallbladder cancers discovered on IOC during LC and CBDE and one with Type IV Mirizzi Syndrome requiring bilioenteric bypass. All the initial procedures had been concluded laparoscopically and the patients were subsequently referred to specialised units for resection and/or biliary reconstruction.

Three patients had complications requiring remedial open surgery at specialist liver units. Two required biliary reconstruction the day after LC due to iatrogenic CBD injuries. One patient had an exploration and T-Tube placement for a Type III Mirizzi Syndrome and subsequently developed bile leakage and bleeding requiring reoperation and biliary bypass. Two patients with peripheral vascular disease developed acute total mesenteric ischaemia a few days after laparoscopic biliary surgery, and laparotomies revealed non-salvageable total bowel infarction. Two had laparotomies for colonic perforations: one with an iatrogenic injury due to adhesiolysis and one with a coincidental perforation of an undiagnosed colonic tumour, both developed severe sepsis.

Five patients who had CBDE required relaparoscopy for biliary drain-related complications: two for retracted T-tubes, one for difficulty in removing a T- tube, one to replace a faulty friable T-tube and one to remove the remnant of a transcystic tube broken during removal. Other causes are summarised in Table 3.

**Radiologically guided percutaneous drainage** of postoperative abdominal collections was carried out in 17 (0.3%) patients: nine for intra-abdominal abscesses and eight for bile leaks.

### Haemostasis

Five patients (0.1%) required simple suturing of bleeding from port sites under local anaesthesia: three at the right subcostal port, one at the umbilical port and one at the right flank port. Two patients with haematological disorders had bleeding from the omentum and the epigastric port and required relaparoscopy for haemostasis. All were elective cases.

**Reventilation** was necessary for three patients (0.1%) post LC either due to severe chest infections (*n* = 2) or due to hypoxia and confusion following initial extubation requiring reventilation for 24 h. All three patients were emergency admission and all recovered well.

### Readmission rate

29 readmissions occurred in the biliary re-intervention group (25.9%). These resulted from bile leaks in nine patients, retained stone in eight, abdominal collections in four, management of benign stricture in three, T-tube complications in two, abdominal pain in two and one for removal of a retracted drain.

Five 30-day mortalities occurred in this reintervention cohort including the four who suffered mesenteric ischaemia and colonic perforations. Another patient died at a specialist centre from haemorrhage following attempted embolisation of a hepatic artery aneurysm which was suspected during uneventful LC with IOC and subsequently confirmed on CT scan and angiography.

## Discussion

This study has a reintervention rate due to postoperative complications of 1.6% in spite of a high emergency workload, routine cholangiography, the adoption of index admission surgery for all comers and single session management of bile duct stones. Although most reinterventions occurred in patients who had undergone bile duct explorations with or without biliary drainage, the incidence of those directly related to biliary drainage was much lower than in studies advocating primary closure of choledochotomies. Reinterventions following single session bile duct exploration have to be seen in the context of the complications and reinterventions associated with the currently used staged management of bile duct stones. The reintervention rate in our cohort of patients undergoing LC alone was 0.8%.

A comparison between the group of patients needing reinterventions and the rest of the series shows that certain preoperative characteristics have predictive significance, e.g. emergency admission and presentation with jaundice. Certain operative findings during the original procedure such as difficulty grades 4 and 5, a contracted gallbladder, bile duct stones necessitating bile duct exploration and the need for open conversion also have significant associations with reintervention (Table 4).

**Table 4:** Demographic, preoperative and operative data of patients requiring postoperative reintervention compared to the rest of series

Characteristics	Reinterventions (*n* = 112) (%)	No reinterventions (*n* = 5627) (%)	*P* Value	OR (95% CI)
Gender			*p* < 0.0023	1.80 (1.22–2.65)
Male	43 (38.4%)	1444 (25.7%)		
Female	69 (61.6%)	4173 (74.2%)		
Emergency admission	76 (67.9%)	2478 (44.0%)	*p* < 0.00001	2.68 (1.80–4.00)
Primary admission diagnosis				
Jaundice	40 (35.7%)	897 (15.9%)	*p* < 0.00001	2.93 (1.98–4.34)
Chronic biliary colic	33 (29.5%)	3067 (54.5%)	*p* < 0.00001	0.35 (0.23–0.53)
Cholangitis	12 (10.7%)	115 (2.0%)	*p* < 0.00001	5.75(3.07–10.76)
Operative difficulty grade				
Grade 1	13 (11.6%)	1885 (33.5%)	*p* < 0.00001	0.26 (0.15–0.47)
Grade 2	26 (23.2%)	1717 (30.5%)	*p* = 0.0962	0.69 (0.44–1.07)
Grade 3	21 (18.8%)	1132 (20.1%)	*P* = 0.7206	0.92 (0.57–1.48)
Grade 4	40 (35.7%)	784 (13.9%)	*p* < 0.00001	3.43 (2.31–5.09)
Grade 5	12 (10.7%)	101 (1.8%)	*p* < 0.00001	6.57(3.50–12.33)
CBD exploration	73 (65.2%)	1245 (22.1%)	*p* < 0.00001	6.59 (4.44–9.77)
Gallbladder condition				
Acute cholecystitis	5 (4.5%)	306 (5.4%)	*p* = 0.6521	0.81 (0.33–2.01)
Chronic cholecystitis	47 (42.0%)	3862 (68.6%)	*p* < 0.00001	0.33 (0.23–0.48)
Empyema	12 (10.7%)	397 (7.1%)	*p* = 0.1361	1.58 (0.86–2.90)
Contracted	21 (18.8%)	624 (11.1%)	*p* = 0.0110	1.85 (1.14–3.00)
Hartmann’s pouch stone	6 (5.4%)	41 (0.7%)	*p* < 0.00001	8.01 (3.33–19.30)
Mucocoele	10 (8.9%)	288 (5.1%)	*p* = 0.0719	1.82 (0.94–3.52)
Conversion to open	5 (4.5%)	23 (0.4%)	*p* < 0.00001	11.39 (4.25–30.52)
Length of surgery, median (range)	120 (32–390) min	60 (15–630) min		
Mortality	5 (4.5%)	3 (0.1%)	*p* < 0.00001	87.60 (20.67–371.26)
Length of hospital stay, median (range)	16 (1–160) days	4 (1–100) days		
Readmissions	27 (24.1%)	153 (2.7%)	*p* < 0.00001	11.36 (7.16–18.04)

A systematic review was conducted in 2018 including 233 studies to identify range and consistency of definition of reported complications following LC. The study found open conversions to be the most common reported complications (58% of studies) followed by bile leakage in 38% and bile duct injury in 32%. 42% of the included studies reported reinterventions following LC [9]. This demonstrates that despite LC being the standard treatment for gallstone disease, further reintervention for postoperative complications is occasionally required and should, therefore, be included in the consent process.

Although the current study had a high workload of biliary emergencies and subsequently a high percentage of difficult cholecystectomies, the morbidity rates for the whole study [6] were lower than many published studies [10–16]. Laparoscopic CBDEs were also performed in the emergency cohort with a low reintervention rate compared to other studies on CBDE [11–18] as shown in Table 5.

**Table 5:** Comparison of postoperative reintervention for complications in studies reporting laparoscopic bile duct exploration. P/C- percutaneous drainage

Study year			Aawsaj ^(11)^ ^2016^	Martin^(16)^ ^1998^	Vidagany^(12) 2016^	Jameel^(13)^ ^2008^	Santo^(14)^ ^2012^	Lauter^(15)^ ^2000^	Tan^(36)^ ^2010^	Rhodes^(37) 1998^	Current study
Total patients			296	300	160	71	57	51	45	40	1318
Bile duct exploration technique	Transcystic		63	173	–	9	26	26	27	28	870
	Failed transcystic converted to choledochotomy		22	–	–	-	11	–	–	–	–
	Choledochotomy		211	127	160	62	20	25	18	12	448
Pre-operative ERCP			17	22	37	12	5	–	28	8	90
T-tube			15	61	–	12	14	24	8	12	226
Clearance rate			84.8%	90.0%	96.2%	88.7%	78.9%	85.0%	86.0%	75.0%	97.9%
Morbidity rate			13.5%	7.3%	15.0%	16.9%	11.8%	19.7%	8.0%	17.5%	18.7%
Reintervention rate			17.6%	12.0%	10.6%	7.0%	15.8%	17.6%	17.8%	25.0%	6.1%
Reintervention post transcystic bile duct explorations		ERCP	–	16	–	3	8	3	2	5	15
		Relaparoscopy	–	3	–	–	–	–	–	–	3
		Laparotomy	–	3	–	–	–	1	–	–	-
		Others	–	–	–	–	–	–	1 P/C	–	5 P/C
Reintervention post choledochotomy bile duct exploration		ERCP	48	9	8	–	–	4	5	4	32
		Relaparoscopy	4	5	6	2	1	–	–	–	9
		Laparotomy	–	–	–	–	–	–	–	1	7
		Others	3 haemostasis	–	3 P/C	–	–	1 intervention radiology	–	–	2 gastroscopy 1 chest drain 3 P/C 1 reventilation

The benefits of delivering an emergency biliary service in this study are reflected in a low reintervention rate for cholecystectomies done without bile duct explorations. In a cohort of 32,785 LCs, Ros et al. [1] reported a significantly higher incidence of 1% reinterventions during readmissions within one year of discharge between 1992 and 1995. In addition, reoperations post bile duct surgery, reconstruction or anastomosis between the bile duct and gastrointestinal tract, and reoperation for unspecified reasons were found increased upon the introduction of LC. There was a tenfold increase in endoscopic and percutaneous reinterventions compared to open cholecystectomy. However, the data were collected from 1987 to 1995 when most surgeons performing LC were in the learning phase of the new technique.

In the current study, where IOC is routinely performed, less than 1.6% of the patients who underwent LC with or without CBDE had complications requiring reintervention including postoperative ERCP in only 1.1% of the series. On the other hand, Ragulin-Coyne et al. [19] found that routine IOC was associated with a higher rate of complications and additional cost. However, their complications were mainly due to post-operative infections (4.3%) and the additional reintervention procedures (included ERCP in 15.8% and CBDE in 2.6%). It is likely, therefore, that routine IOC ± CBDE, as would be the case in a specialised unit, would have prevented most of these reinterventions. Li J et al. [20] suggested that where routine IOC was performed, one ERCP was avoided for every 18.5 IOCs performed.

Preoperative ERCP remains the main method of clearing CBD stones in most units. However, our results showed that one session laparoscopic management of biliary stones is associated with lower morbidity than published results of ERCP, a finding which was consistent with several studies [18–23]. Kadam et al. [21] reported lower morbidity for LC and CBDE, with bile leakage occurring in 6.7%, while two-stage ERCP/ stenting + LC was associated with 16.7% pancreatitis and 6.7% cholangitis. There was a high rate of reintervention in the ERCP group with 2.3 procedures per patient. A systematic review by Kenny et al. [22] concluded that a single-stage approach to managing symptomatic gallstones and CBD stones is the preferred procedure. Rogers et al. [23] prospectively randomised LC and laparoscopic CBDE against ERCP and found that CBDE eliminated the risk of post-ERCP pancreatitis and the need for further procedures. Gupta et al. [24] also found that ERCP involved more reinterventions than one-stage treatment of bile duct stones. Laparoscopic CBDE also reduces the incidence of recurrent stones, thus avoiding inevitable future reinterventions [25, 26].

In spite of its benefits, biliary drainage is still a main cause of morbidity following bile duct exploration. In this series of 5740 LC, 527 (9.2%) had CBDE with biliary drainage (transcystic or T-tubes) resulting in 6 reinterventions (1%): 5 relaparoscopies and 1 ERCP. However, biliary drainage following choledochotomy helped to minimise the risk of bile leakage, and to obtain postoperative cholangiography. In this study biliary drains were used to clear a few retained stones, flushing the bile duct after Glucagon administration and to dissolve blood clots in the bile duct using Alteplase, a fibrinolytic agent, thus avoiding invasive reinterventions. The use of the T-tube track to remove retained stones was first reported in 1978 by Burhenne [27], but endoscopic reinterventions have reduced the reliance on this technique. On the other hand biliary drains can result in complications. Gillatt et al. [28] reported bile leakage upon removal of the T-tube in 18% but only one of 39 patients required reoperation due to bile peritonitis. Sharma et al. [29] reported one patient (2.5%) and Wills et al. [30] had T-tube complications in 15.3% with a quarter requiring surgical reinterventions. Biliary drain complications are thus associated with a low reintervention rate reflecting the fact that most such complications can be managed conservatively.

12 patients (0.02%) in this series were found to have pancreatic, periampullary or gallbladder cancers that required postoperative reintervention. Eight needed ERCP for stenting and 4 underwent Whipple’s procedures. In systematic reviews, unsuspected gallbladder cancer was found by Choi et al. [31] in 0.7% and by Soreide et al. [32] in 0.25%–0.89% routine LCs.

The incidence of bile duct injury varies in the literatures [31–35]. In the current study, two CBD injuries (0.03%) occurred and were identified intraoperatively. IOC outlined the biliary anatomy and helped to avoid making the injury worse. Biliary stents and abdominal drains were inserted and the patients were referred to a tertiary liver surgery unit where uneventful biliary reconstruction was carried out within 24 h. Tornqvist et al. [36] quoted an incidence of bile duct injury between 0.2% and 0.9%, while Viste et al. [37] reported a frequency of 0.4% of main bile duct injury. The low biliary injury rate seems to have been contributed to, at least in part, by routine IOC, which in the context of a specialised service is safe in all elective and emergency patients.

## Conclusion

Reintervention resulting from complications occurred in only 1.6%, in spite of the policy of index admission surgery for all comers including emergency admissions and single session management of bile duct stones. Surgical or endoscopic reinterventions following LC alone occurred in 0.8%. Some preoperative and operative characteristics are predictive of reintervention and may guide preventive strategies. The most common form of reintervention was ERCP for retained CBD stones. This important outcome parameter of laparoscopic biliary surgery can be optimised through early diagnosis and timely reintervention for complications.
